# A dual deep neural network for auto-delineation in cervical cancer radiotherapy with clinical validation

**DOI:** 10.1186/s13014-022-02157-5

**Published:** 2022-11-15

**Authors:** Shihong Nie, Yuanfeng Wei, Fen Zhao, Ya Dong, Yan Chen, Qiaoqi Li, Wei Du, Xin Li, Xi Yang, Zhiping Li

**Affiliations:** 1 Department of Radiotherapy, Cancer Center, West China Hospital, Sichuan University, No. 37 Guo Xue Alley, Chengdu, 610065 People’s Republic of China; 2 Department of Oncology, Chengdu First People’s Hospital, No. 18 Wanxiang North Road, Chengdu, 610041 People’s Republic of China

**Keywords:** Cervical cancer radiotherapy, Clinical target volume auto-segmentation, Organs-at-risk auto-segmentation, Artificial intelligence-assisted system

## Abstract

**Background:**

Artificial intelligence (AI) algorithms are capable of automatically detecting contouring boundaries in medical images. However, the algorithms impact on clinical practice of cervical cancer are unclear. We aimed to develop an AI-assisted system for automatic contouring of the clinical target volume (CTV) and organs-at-risk (OARs) in cervical cancer radiotherapy and conduct clinical-based observations.

**Methods:**

We first retrospectively collected data of 203 patients with cervical cancer from West China Hospital. The proposed method named as SegNet was developed and trained with different data groups. Quantitative metrics and clinical-based grading were used to evaluate differences between several groups of automatic contours. Then, 20 additional cases were conducted to compare the workload and quality of AI-assisted contours with manual delineation from scratch.

**Results:**

For automatic CTVs, the dice similarity coefficient (DSC) values of the SegNet trained with incorporating multi-group data achieved 0.85 ± 0.02, which was statistically better than the DSC values of SegNet independently trained with the SegNet^(A)^ (0.82 ± 0.04), SegNet^(B)^ (0.82 ± 0.03) or SegNet^(C)^ (0.81 ± 0.04). Moreover, the DSC values of the SegNet and UNet, respectively, 0.85 and 0.82 for the CTV (P < 0.001), 0.93 and 0.92 for the bladder (P = 0.44), 0.84 and 0.81 for the rectum (P = 0.02), 0.89 and 0.84 for the bowel bag (P < 0.001), 0.93 and 0.92 for the right femoral head (P = 0.17), and 0.92 and 0.91 for the left femoral head (P = 0.25). The clinical-based grading also showed that SegNet trained with multi-group data obtained better performance of 352/360 relative to it trained with the SegNet^(A)^ (334/360), SegNet^(B)^ (333/360) or SegNet^(C)^ (320/360). The manual revision time for automatic CTVs (OARs not yet include) was 9.54 ± 2.42 min relative to fully manual delineation with 30.95 ± 15.24 min.

**Conclusion:**

The proposed SegNet can improve the performance at automatic delineation for cervical cancer radiotherapy by incorporating multi-group data. It is clinically applicable that the AI-assisted system can shorten manual delineation time at no expense of quality.

**Supplementary Information:**

The online version contains supplementary material available at 10.1186/s13014-022-02157-5.

## Introduction

Cervical cancer is the second most common type of malignant tumor that poses a threat to women’s health [1], and it has the fourth highest incidence among any type of cancer in women worldwide, in addition to being the most frequent gynecological cancer in many developing countries [2, 3]. Current clinical treatments for cervical cancer primarily include surgery, radiotherapy, and chemotherapy [4] and of these, radiotherapy has a 5-year survival rate of 87–92% for the treatment in women with stage IB cervical cancer [5]. There are several ways to perform cervical cancer radiotherapy, with photon radiation being the most common approach, such as intensity-modulated radiation therapy (IMRT). IMRT can deliver a relatively large radiation dose to the clinical target volume (CTV) and reduce the radiation dose to adjacent organs-at-risk (OARs), thereby effectively reducing the postoperative local recurrence rate of cervical carcinomas and providing better protection for non-cancerous tissues [6, 7]. It was reported that manual contouring of the CTV and OARs on a patient’s computed tomography (CT) scans is time-consuming and labor-intensive [8]; on the other, CTV contouring has large inter-and intra-observer variation among radiation oncologists with different levels of clinical experience [9–11]. Therefore, the development of quick and effective computer-aided tools to automatically delineate the region of interest (ROI) can reduce the manual delineation workload and ensure quality between radiation oncologists with difference working experience.

Over the past decade, since deep convolutional neural networks (DCNNs) can automatically learn task-related features in a data-driven manner [12, 13], artificial intelligence (AI) algorithms have been developed for a variety of applications in medical image analysis [14–16]. Recently, in cervical cancer radiotherapy, Liu et al. [17]described a two-dimensional UNet [18] for segmentation of the OARs of 105 patients, and Sartor et al. [19]developed a fully convolutional three-dimensional (3D) model [20] for segmentation of the CTV and OARs of 75 patients. Liu et al. further developed a 2.5D model for cervical cancer radiotherapy of 210 patients and validated it performance of 27 patients [21]. Most relevant research has focused on the performance of automatic results based on a structural dataset. However, few studies have been concerned with the following challenges: (1) although there are international guidelines for the delineation and treatment of cervical cancer [22], CTV contouring still has large variation, which can lead to diversity of the collected data. The influence of multi-group dataset using the data-driven deep convolutional neural networks has not been investigated; (2) previous subjective evaluations of automatic contours hasn’t provided objective clinical qualitative criteria; (3) Insufficient testing data may fail to generate reliable evaluation [23–25]; (4) previous clinical validation observations mainly focus on the time [17, 26]. To deal with the above problems, more efforts should be dedicated to constructing datasets with multi-group cases and considering the influence of the data on AI algorithms employed. A large number of testing set is required for clinical-based observational study and comparable experiments are conducted on the time and quality between radiation oncologists with difference working experience.

In this study, we first constructed a relatively large cervical cancer dataset of 203 patients including three groups with retrospectively collected manual annotations, and of these, 60 cases were used for clinical-based analysis. The evaluations based on such a testing set would be more reliable. Second, we developed specific deep convolutional neural networks for automatic contouring of the CTV and OARs of the constructed dataset. The results of AI models trained with different data groups were also evaluated. Finally, we integrated deep convolutional neural networks to implement the AI-assisted system for automatic delineation, and several doctors with different experience validated the system on 20 additional cases.

## Material and methods

### Study design and participants

This study was designed to develop deep convolutional neural networks for automatic contouring of the CTV and OARs on cervical cancer CT images. The definition of the CTV was based on the consensus guideline [22], and the contoured OARs were the bowel bag, left and right femoral heads, bladder, and rectum. This study was approved by the Institutional Ethics Review Board of West China Hospital, Sichuan University and waived informed consent.

Between February 2018 and April 2020, the CT images of 203 patients with pathologically proven stage IA1–IB2 cervical cancer who were treated with post-operation radiotherapy were retrospectively collected from three groups led by three senior radiation oncologists in our department of West China Hospital, Sichuan University. The inclusion criteria were: (1) patients with pathologically proven stage IA1–IB2 cervical cancer, (2) who were treated with post-operation radiotherapy, (3) CT scan for positioning, (4) could obtain the CT images. The exclusion criteria were: (1) patients with cervical cancer who are not candidates for radiation therapy, (2) patients with advanced cervical cancer. Specifically, 71, 67, and 65 cases were collected from the three groups led by three senior radiation oncologists, respectively. In the clinical routine, the annotations of the CTV and OARs on CT images in each group were first manually delineated by junior oncologists, and then reviewed and approved by other leading experienced oncologists. Moreover, the anonymized data set consists of 203 patients’ CT images were reconstructed with 3 mm thickness and 0.9 m × 0.9 m in-plane resolution using a GE Revolution ES CT scanner. The patient was in the supine position during CT scans. Before the CT scans, bladder and rectal preparation were performed. We did not use the contrast agent for the bladder filling. Then the 203 patients were randomly divided into three sets, 121 cases of which were randomly selected for training set, 22 cases for validation set, while the remaining 60 cases were testing set in a ratio of approximately 6:1:3. To further evaluate the practical value of the AI-assisted system, 20 additional cases were prospectively recorded and analyzed from August 2020 to November 2020.

### Development of deep convolutional neural network models for automatic contouring

In this study, the automatic contouring procedure was implemented in a two-stage method called SegNet. The first stage distinguishes slices of interest from all slices of continuous 3D CT scans. The continuity of these interested slices containing ROIs is essential for the following delineation of ROIs. The second stage is a segmentation task based on the results of stage 1. The proposed SegNet took CT slices as input, and the corresponding automatic contours were calculated as output. We used a dense convolutional network (DenseNet) [27]for the first identification task and a novel encoder-decoder network for the segmentation. The encoder of SegNet consists of residual convolutional blocks [28], and densely connected blocks were used as the backbone of the decoder. SegNet was developed based on UNet by introducing shortcut connections and deeper convolutional layers. The frameworks of SegNet are shown in Additional file 1: Fig. S1. The detailed process and architecture of the two-stage method are given in Additional file 1: Appendix 1.

### Quantitative and qualitative evaluation

For objective evaluation, we used sensitivity and area under curve (AUC) to show the recognition accuracy of the first stage identification task. Higher scores represent better continuity of slices. Three widely used quantitative metrics were adopted for the final evaluation of ROI contouring: the volumetric dice similarity coefficient (DSC) [29], the 95% Hausdorff distance (95HD) [30], and the true positive volume fraction (TPVF) [31].

The automatic CTV contours created on the testing set were assessed clinically. A six-point set of objective evaluation criteria was designed following the international guideline [22], as shown in Table 1. The resulting contours of AI models trained with whole multi-group dataset were recorded as SegNet and UNet, and the automatic contours of the same architecture SegNet only trained with a single group data (A, B or C) were called as SegNet^(A)^, SegNet^(B)^, and SegNet^(C)^, respectively. Three radiation oncologists independently graded these automatically segmented CTVs. The score for each case was either 0 or 1: 0- failing the criteria; 1-reaching the criteria. If all the 6 target sites were achieved the criteria in one patient, 6 points were given, and a full score of 60 patients was recorded as 360 points. To avoid bias, each radiation oncologist performed the evaluation of automatic CTVs from each model every other day in a randomized double-blind manner. The final qualitative score for each case was the rounded average score of three experts.

**Table 1:** A six-point evaluation criterion for the clinical target volume (CTV) delineation in cervical cancer radiotherapy

Nos.	Criterion	Qualitative evaluation (60 cases)				
		UNet	SegNet	SegNet^(A)^	SegNet^(B)^	SegNet^(C)^
1	The common iliac lymph nodes area is included	56	60	60	59	60
2	The internal iliac lymph nodes area is included	59	60	60	60	60
3	The external iliac lymph nodes area is included	52	59	57	58	55
4	The presacral lymph nodes area is included	40	60	52	50	43
5	The paravaginal tissue area is included	33	53	45	46	42
6	The upper vagina is included	60	60	60	60	60

Main differences between UNet and SegNet rely on different network architectures. The differences between SegNet, SegNet^(A)^, SegNet^(B)^ and SegNet^(C)^ are the scale of the training set, which is 121 (multisource dataset), 42, 40 and 39, respectively

### Testing of the AI-assisted system in clinical setting

The proposed SegNet were integrated to develop an AI-assisted system for automatic contouring of ROIs in cervical cancer radiotherapy. The software has been assessed in the Department of Radiotherapy in West China Hospital since August 2020. The detailed running process and workflow of the AI-assisted system was summarized as follows: the proposed two-stage model was integrated into an artificial intelligence (AI)-assisted system that can be used for automatic delineation of the clinical target volume and organs at risk in the cervical cancer radiotherapy treatment. In general, the workflow of the AI-assisted system consists of the following stages:

- Step 1: Data transfer. The AI-assisted system has a user interface. Radiologists log into the system with their username and password and then select cases for treatment planning. The software sends a request to retrieve the patient’s CT scans from the PACS system.
- Step 2: Automatic delineation. All slices are pre-processed and then used as the inputs to the first stage. Based on the first model’s results, slices likely to contain regions of interest (ROIs) are used as the inputs to the second stage to determine the ROI boundaries. This automatic contouring process does not require any human assistance and eliminates the drawbacks of inter-and intra-delineation variation within the same case.
- Step 3: Manual correction. The AI-generated contours are automatically stored in the Ray Station treatment planning system, on which oncologists can directly re-edit the AI-generated ROI boundaries until the plan has been approved.

In the second step in the workflow, SegNet is able to generate the automatic contours of the CTV and OARs for one case in 13.08 s (on a Linux system with 24 GB of RAM and Nvidia RTX 3090 GPUs), and the AI system’s average time to process a case was approximately 2 min, consisting total three-step workflow.

To analyze the potential value of the AI-assisted system, three radiation oncologists with different clinical experience conducted comparative experiments on 20 new patients who were not included in the development cohort. First, each doctor’s manual revision time of the AI-assisted contours was recorded. The doctors’ time to manually contour the same cases from scratch was recorded after 2 weeks. Moreover, all annotations by the three radiation oncologists were finally reviewed by ZP.L. with more than 30 years of clinical experience to evaluate the quality of the radiotherapy planning according to a 2—grade score: 0—secondary revision (the treatment planning should be re-edited to some extent), or 1—minor or no revision (the planning is basically acceptable for clinical radiotherapy treatment). This comparison was developed to assess the potential influence of the AI-assisted results on radiation oncologists’ plan making. If one patient does not need to modify all six target areas, the score is 6 points, and the full score of 60 patients is 360 points.

### Statistical analysis

All statistical comparisons were performed using SPSS software. The patient characteristics of age were statistically analyzed, statistical analysis of significant differences in age between the training set, validation set and testing set were performed by the chi-square test. DSC, TPVF and 95HD were computed for all the target regions. The independent sample t-test method was used to compare DSC, TPVF and 95HD between SegNet and UNet. The time used for revising all the CTV and OARs’ contours before radiotherapy planning were recorded as minutes per case. Statistical significance was set at two-tailed P < 0.05.

## Results

Supplemental Table S1 shows the characteristics of the patients in this study with statistical analysis. No significant differences were found regarding age or number of cases in the routine groups between the development cohort of the training, validation, and testing set.

The testing set was more heterogeneous than the others because it contained 9848 slices from 60 cases, which impart more reliability to the comparative observations. The sensitivity and receiver operating characteristic (ROC) curves of the first stage automatic identification results are shown in Additional file 1: Fig. S2. The bladder had the highest sensitivity score (0.9875), followed by the CTV (average score: 0.965). The bladder also had the highest area under curve (0.998), followed by the femoral heads (0.997) and the rectum (0.993). The high scores of these automatic identification results indicated good continuity of slices that contained interested ROIs (> 0.95).

The quantitative evaluation of automatic CTVs and OARs by SegNet and UNet trained with whole multi-group dataset is summarized in Table 2. For the CTV, the average volumetric DSC scores predicted by SegNet and UNet were 0.85 and 0.82, respectively, and the mean TPVF values of SegNet and UNet were 0.87 and 0.77, respectively. These differences between the two methods were statistically significant (*P* < 0.001). SegNet also achieved better results than UNet on the contouring of OARs. For the rectum and bowel bag, SegNet showed a significant improvement in DSC and 95HD scores over UNet. For instance, for the bowel bag, volumetric DSC for SegNet and UNet were 0.89 and 0.84, respectively; 95HD for SegNet and UNet were 9.95 mm and 18.78 mm, respectively. Besides, TPVF scores for the bladder and the right and left femoral heads predicted by SegNet were 0.95, 0.92, and 0.93, respectively. In Table 1, the grading of SegNet’s results was greatly different from that of UNet, especially in terms of the region of the presacral lymph nodes and paravaginal tissue (Additional file 1: Fig. S3). Overall, more than 98% (352/360) predicted by SegNet were totally clinically acceptable, whereas the evaluation score for UNet was 83% (300/360).

**Table 2:** Volumetric Dice similarity coefficient (DSC), true positive volume fraction (TPVF), and 95% Hausdorff distance (95HD) scores of the six automatic contours predicted by SegNet and UNet

	Volumetric DSC			TPVF			95HD		
	SegNet	UNet	*P*	SegNet	UNet	*P*	SegNet	UNet	*P*
CTV	0.85 ± 0.02	0.82 ± 0.03	< 0.001	0.87 ± 0.04	0.77 ± 0.07	< 0.001	7.91 ± 2.93	9.44 ± 4.53	0.03
Bladder	0.93 ± 0.05	0.92 ± 0.05	0.44	0.95 ± 0.04	0.90 ± 0.07	< 0.001	6.83 ± 8.79	6.04 ± 5.52	0.54
Rectum	0.84 ± 0.06	0.81 ± 0.06	0.02	0.84 ± 0.09	0.84 ± 0.09	0.72	8.81 ± 7.04	13.20 ± 12.01	0.02
Bowel bag	0.89 ± 0.04	0.84 ± 0.05	< 0.001	0.88 ± 0.05	0.79 ± 0.07	< 0.001	9.95 ± 5.45	18.78 ± 23.55	0.01
R-FH	0.93 ± 0.04	0.92 ± 0.05	0.17	0.92 ± 0.07	0.88 ± 0.08	0.01	4.58 ± 7.01	5.25 ± 7.28	0.61
L-FH	0.92 ± 0.03	0.91 ± 0.04	0.25	0.93 ± 0.07	0.90 ± 0.07	0.027	4.71 ± 4.65	6.83 ± 17.96	0.38

The performance of two methods was tested by the independent sample t-test method. R-FH is short for the right femoral head. L-FH is short for the left femoral head

**Fig. 1:**
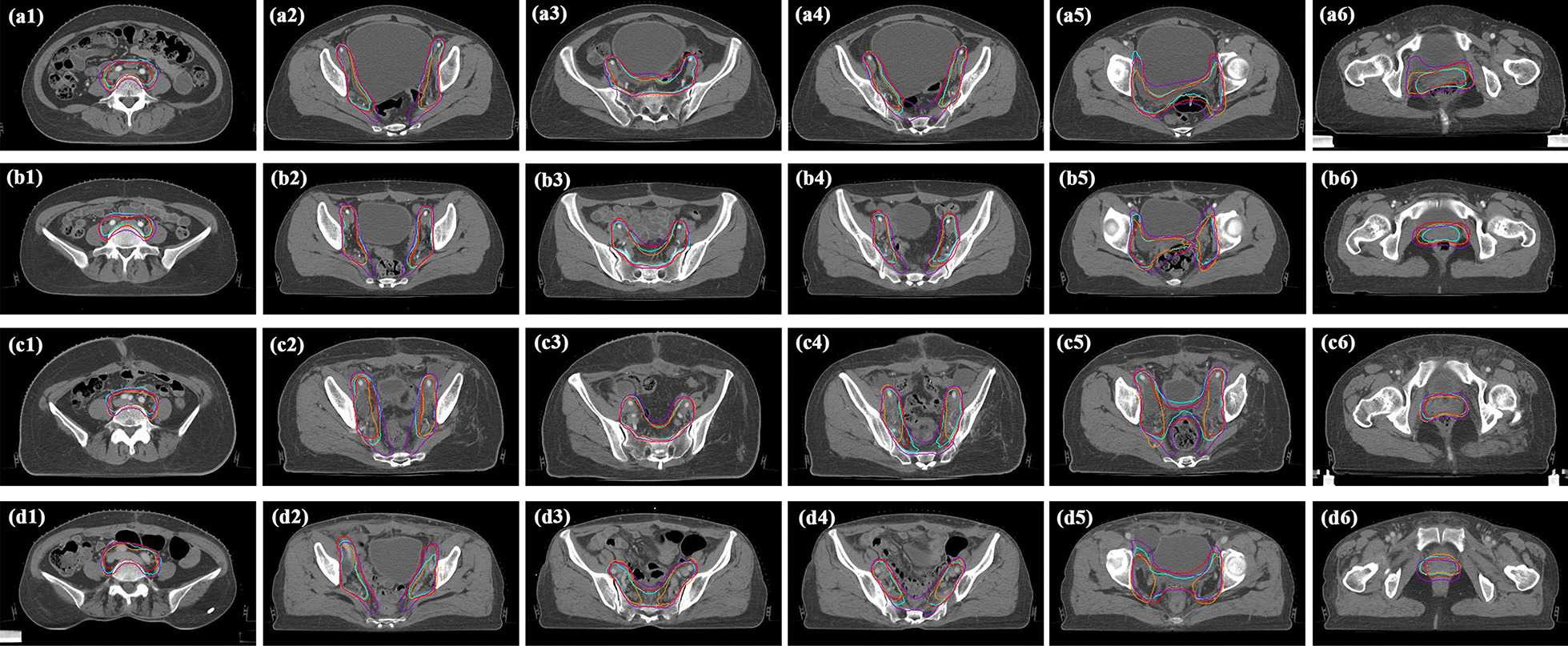
Clinical target volume (CTV) contouring predicted by SegNet^(A, B, C)^. The orange, turquoise, purple, and red contours represent the CTV segmented by SegNet^(A)^, the CTV by SegNet^(B)^, the CTV by SegNet^(C)^, and the corresponding manual annotations, respectively. The first and second columns indicate the areas of the common iliac lymph area and the internal and external iliac lymph nodes, respectively. The third and fourth columns present the presacral lymph nodes area and the end of this area, respectively. The fifth and sixth columns indicate the paravaginal tissue and upper vagina areas, respectively. In Case **a**, the volumetric DSC quantitative scores by SegNet^(A)^, SegNet^(B)^, and SegNet^(C)^ were 0.81, 0.86, and 0.82, respectively. In cases **b**–**d**, the volumetric DSC scores for SegNet^(A)^, SegNet^(B)^, and SegNet^(C)^ were 0.87/0.84/0.81, 0.80/0.86/0.85, and 0.80/0.86/0.82, respectively

The quantitative evaluation of the automatic CTVs generated by SegNet ^(A)^, SegNet ^(B)^ and SegNet ^(C)^ independently trained by the single group are summarized in Table S2. The mean DSC and 95HD values of SegNet^(A)^, SegNet^(B)^, and SegNet^(C)^ were 0.82, 0.82, and 0.81 and 10.33 mm, 9.57 mm, and 10.42 mm, respectively. However, as clinical grading listed in Table 1, SegNet^(C)^ had the worst clinical-based score. The differences in automatic CTVs among three models mainly occurred in the end of the presacral area (Fig. 1, fourth column). As for criterion 4, among the 60 graded cases, SegNet^(A)^ and SegNet^(B)^ passed 52 and 50 cases, whereas SegNet^(C)^ only passed 43 cases.

The times of manual contouring from scratch and AI-assisted revision of only CTV contouring are shown in Table 3. The average time for three doctors was 30.95 ± 15.24 min. By comparison, the average time taken to manually revision of AI-assisted contours was 9.54 ± 2.42 min. The number of plans approved after using the AI-assisted system was slightly larger than those with manual delineation from scratch.

**Table 3:** Comparisons time between artificial intelligence (AI)-assisted CTV correction and manual CTV contouring for 20 additional cases

	Manual contouring from scratch			AI-assisted correction		
	Doctor 1	Doctor 2	Doctor 3	Doctor 1	Doctor 2	Doctor 3
Time (/min)						
Range	15.67–22.28	22.27–42.13	36.03–38.21	5.07–9.37	7.55–15.85	8.70–17.48
Median (IQR)	18.09 (17.43–19.07)	33.03 (25.62–38.14)	38.21 (36.03–43.98)	7.37 (6.47–7.88)	9.90 (8.36–10.58)	11.16 (9.73–12.48)
Number of approving cases (n = 20)	16	17	16	16	18	18

Doctor 1 has more than 5 years of working experience in cervical cancer radiotherapy. Doctor 2 and doctor 3 have about 3 years of experience

The key raw data and the system demo have been uploaded to github (https://github.com/luvWY/AutomaticContouring.git).

## Discussion

It was clinically acceptable to apply the proposed SegNet to automatically delineate the CTV and OARs in cervical cancer radiotherapy. Additionally, the experimental results showed that training the SegNet using data from multi-group dataset can improve the quality of the automatic contouring and achieve more robust performance. To the best of our knowledge, this was the first study that provided clinical evaluations on an adequate number of test set cases by several methods, developed a clinically applicable AI-assisted system, and compared the quality and time cost between AI-assisted results and manual delineation (20 cases).

**Fig. 2:**
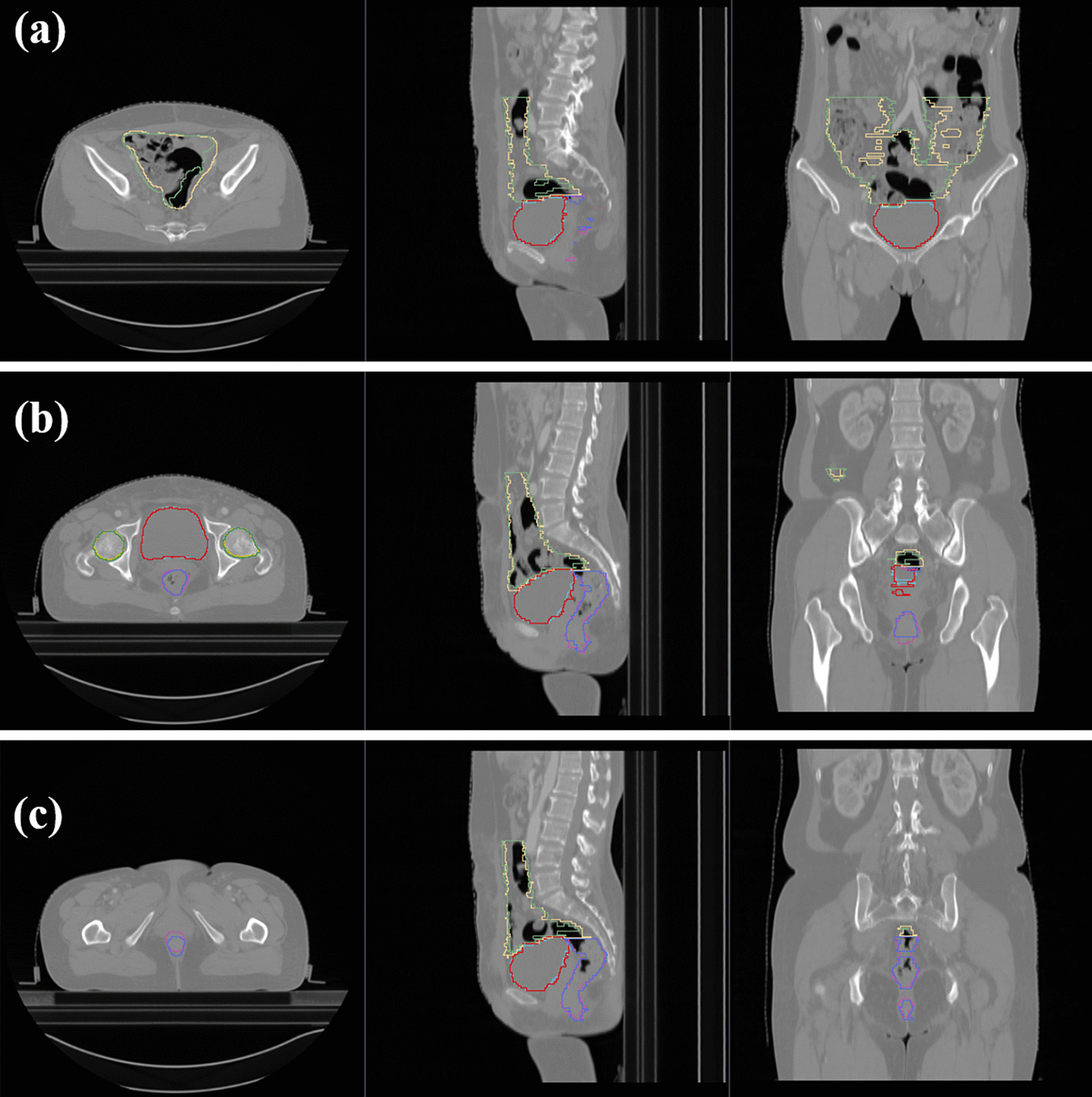
Automatic contours for organs-at-risk predicted by SegNet. Parts **a**–**c** are the upper, middle, and lower three-dimensional sections, respectively. The lemon green and lemon yellow areas indicate the automatic bowel bag contours and the manual annotation, respectively. The blue and red areas denote the automatic bladder contours and the corresponding labels, respectively. The dark green and dark yellow areas indicate the automatic contours for the left and the right femoral head and the labels, respectively. The purple and the violet blue contours indicate the automatic rectum results and the manual annotations, respectively. The quantitative DSC results for the bladder, rectum, bowel bag, and left and right femoral head were 0.96, 0.90, 0.90, 0.94, and 0.93, respectively

The 60 cases in the testing set were evaluated independently. Both of deep convolutional neural networks can detect the obvious visual edges of OARs such as the femoral heads and the bladder to achieve a high-quality score. The average volumetric DSC and TFVF scores of the femoral heads and bladder were greater than 0.9, which greatly reduced the workload associated with manual correction of these organs. However, the contouring quality of the rectum and bowel bag using the deep convolutional neural networks was slightly lower. The reason might be that the boundary between the lower rectum and the surrounding soft tissue was less obvious because of CT’s low resolution in soft tissue and the low-density difference between rectum and soft tissue. Moreover, because the abdominal cavity is large, there was a large variation in cavitary organs among different individuals. For example, the boundary of the bowel bag for a patient with intestinal inflation should include the gas area, but the automatic results failed to cover this area (Fig. 2a). These problems indicate that the automatic results learned in a data-driven manner from a limited dataset may lead to poor performance in some certain aspects, especially for unclear boundaries.

The quantitative metrics of the predicted CTV by SegNet were also superior to those by UNet. The grading results also showed that automatic delineation results predicted by SegNet were more clinically acceptable than those by UNet. In terms of criteria 1, 2, 3, and 6, the predicted results from both two models were highly consistent with the cervical contouring guideline, while the differences between two methods were large in terms of criteria 4 and 5. For instance, as shown in the fifth column of Additional file 1: Fig. S3, the automatic CTVs from SegNet completely covered the parametrial area, whereas the prediction by UNet was inconsistent with the manual annotation. It indicated SegNet performed better at detection of targets with more indistinct boundaries since it had deeper network architecture and shortcut connections to facilitate learning of task-related feature representations.

In Table 1, three radiation oncologists concluded that the overall performance of SegNet trained by multi-group data of 121 cases was the best. The number of training cases for SegNet^(A)^, SegNet^(B)^ and SegNet^(C)^ are nearly equal, while the performance of SegNet^(C)^ was significantly worse than that of the other two models (SegNet^(A)^ and SegNet^(B)^). The reason for this finding may be that the training datasets played a crucial role in developing deep convolutional neural networks. Since the training of AI algorithms is a data-driven manner, the dataset covering various features and variations tend to access more robustness. This finding is basically consistent with the current research findings that mixing different groups or sources for AI algorithms can help to eliminate the influence of the variety and preferences and achieve more clinically satisfactory results [23, 25, 26, 32, 33].

Further analyzing five groups automatic CTVs in Table 1, the clinical results showed that the models’ automatic contouring boundaries of the presacral lymph nodes and the paravaginal tissue area required the most manual correction. For instance, in the delineation of the paravaginal tissue area, the automatic results sometimes failed to cover this area entirely. There could be two reasons for this finding: (1) some high-risk subclinical lesions near the parametrial area are not defined in detail, but they may be clinically important, depending on the doctors’ experience; (2) the parametrial area varies greatly among different individuals. For example, in some patients with a large pelvic cavity, the intestines may fall into the pelvic cavity after surgery and fill the original position of the ovaries and uterus, which leads to complexities and unclear boundaries of the parametrial area. If we can collect more data to ensure the model’s generalization performance, emphasizing these noteworthy features in advance, the deep learning algorithm would be likely to perform better from the clinical perspective.

In terms of delineation time in Table 3, the automatic contouring was proved to be time-saving for radiation oncologists, especially for junior doctors. Furthermore, to resolve the open question of whether automatic predictions influence radiation oncologists’ decision making in comparison with manual delineation alone, the three doctors’ acceptance rates were at the same level or slightly improving. It was indicated that AI-assisted system might be able to improve the junior radiation oncologists’ contouring quality and there was no negative impact in practice. Based on clinical observations, the main difference always occurred in the parametrial area, which could be expected considering its complexity as addressed above. For instance, on the basis of the patient’s other clinical materials, it is possible that the parametrial area should be enlarged to encompass some high-risk subclinical lesions that may lead to local recurrence. In contrast, the same area might contain parts of intestines that are unnecessary to irradiate, and the irradiation of which may aggravate radiation enteritis.

## Conclusion

The AI-assisted system achieved good accuracy at contouring the CTV and OARs in cervical cancer radiotherapy from the clinical perspective, which reduced the workload of manual delineation at no expense of quality. Further studies are necessary to collect multi-center data and validate the AI-assisted system in different centers.

## Supplementary Information

**Additional file 1.** Supplemental methods, tables, and figures.

## Data Availability

The key raw data and the system demo have been uploaded to github (https://github.com/luvWY/AutomaticContouring.git).

## Abbreviations

AI: Artificial intelligence
AUC: Area under curve
CT: Computed tomography
CTV: Clinical target volume
DSC: Dice similarity coefficient
IMRT: Intensity-modulated radiation therapy
OARs: Organs-at-risk
ROI: Region of interest
TPVF: True positive volume fraction
