# Supplementary material for: A dual deep neural network for auto-delineation in cervical cancer radiotherapy with clinical validation
Source: Radiat Oncol. 2022 Nov 15;17:182. doi: 10.1186/s13014-022-02157-5 (PMC9667653; doi:10.1186/s13014-022-02157-5)
Supplement: Supplementary file 1 — Additional file 1. Supplemental methods, tables, and figures. [file 13014_2022_2157_MOESM1_ESM.docx]

**Contents**

[Section 1: Supplemental Methods 2](#_Toc118762656)

[Appendix 1. Description of deep convolutional neural networks 2](#_Toc118762657)

[A. A two-stage computing process 2](#_Toc118762658)

[B. The network architecture 3](#_Toc118762659)

[C. Implementation details 3](#_Toc118762660)

[Section 2: Supplemental Tables 4](#_Toc118762661)

[Table S1 4](#_Toc118762662)

[Table S2 5](#_Toc118762663)

[Section 3: Supplemental Figures 5](#_Toc118762664)

[Figure S1 5](#_Toc118762665)

[Figure S2 6](#_Toc118762666)

[Figure S3 7](#_Toc118762667)

# Section 1: Supplemental Methods

## Appendix 1. Description of deep convolutional neural networks

The proposed two-stage method (SegNet) included the two-phase computing process of independent deep convolutional neural networks with the corresponding network architecture. Further details are described below.

### A. A two-stage computing process

Given a case $X=\{x_{i}$|$i=1,2,\cdots,n\}$, where $n$ is the number of slices (or images) in case $X$, the workflow of the first stage can be formulated as $y_{i}=F1\left( x_{i} \right)$, where $x_{i}$ denotes the $i$-th slice in case $X$, and $y_{i}\in\left\{ 0,1 \right\}$ presents the target volume in terms of whether there are ROIs in $x_{i}$. ‘$y_{i}=1$’ denotes the $i$-th slice containing ROIs of interest. Function $F1(\cdot)$ is the mapping algorithm learned by the first model using the back-propagation algorithm. On the basis of the outputs of the first stage, the slices $x_{i}$ with predictions of 1 were constructed as the inputs to the second segmentation stage. The computing process can be formulated as $y_{i}^{mn}=F2\left( p_{i}^{mn} \right)$, where $p_{i}^{mn}$ is the pixel at location $(m,n)$ in the $i$-th slice ($x_{i}$), and $y_{i}^{mn}\in\left\{ 0,1 \right\}$ is the corresponding label denoting whether there are ROIs at the location $p_{i}^{mn}$. ‘$y_{i}^{mn}=1$’ denotes that location $(m,n)$ in the$i$-th slice was involved in the ROI. Function $F2(\cdot)$ is the mapping result learned by the second model using the deep learning back-propagation algorithm. The default segmentation result was 0 for $y_{i}^{mn}$of$x_{i}$ with the prediction of 0 in the first stage. Finally, the output of a case $X$ is defined as $Y=\{y_{i}^{mn}$|$i=1,2,\cdots,n\}$.

### B. The network architecture

The proposed two-stage model consists of two subnetworks. The first model is based on DenseNet, with the last fully connected layer replaced by binary neurons. The full architecture mainly consists of 169 layers including dense blocks and transition layers. The second model is the typical encoder-decoder architecture. The encoder was constructed using the ResNet architecture with 152 layers, and the decoder consists of three densely connected blocks and upsampling. The encoder was used as a feature extractor on the input, and the final predictions of the corresponding region of interest were then generated by the decoder.

### C. Implementation details

The training process of the proposed model mainly consisted of two phases: data pre-processing and network learning. In the data pre-processing phase, the pixel values for each CT slice were truncated to the HU range [−800, 1400] and normalized between 0 and 1. In the first stage, the CT images were resized from 512 × 512 × 3 to 224 × 224 × 3, and the input shape of the second stage remained 512 × 512 × 3. The back-propagation algorithm was used for network learning. All the network’s learnable parameters were first initialized on ImageNet dataset then fine-tuned by deploying the back-propagation algorithm only on the training set. All models were trained independently on the corresponding dataset and implemented using the PyTorch deep learning framework.

# Section 2: Supplemental Tables

## Table S1

Characteristics of the women who participated in this study.

|  |  | Development cohort (n=203) | | | *P* | Additional testing cases  (n=20) |
| --- | --- | --- | --- | --- | --- | --- |
|  |  | Training set  (n=121) | Validation set  (n=22) | Testing set  (n=60) |  |  |
| Age | Range | 29-71 | 34-71 | 33-71 |  | - |
|  | Median (IQR) | 51 (45-56) | 54 (47-57) | 52 (46-56) |  |  |
|  | <40 year | 11 | 2 | 6 | 0.78 |  |
|  | 40-60 year | 95 | 15 | 46 |  |  |
|  | >60 year | 15 | 5 | 8 |  |  |
| Routine groups | A (n=71) | 42 | 8 | 21 | 1 | 20 |
|  | B (n=67) | 40 | 7 | 20 |  |  |
|  | C (n=65) | 39 | 7 | 19 |  |  |

Note: Age and the distribution in the development cohort were tested by the chi-square test here. Clinical evaluation for the AI-assisted system was based on new patients with no need for recording age and routine groups.

## Table S2

Volumetric DSC, TPVF, and 95HD scores of automatic CTVs predicted by SegNet trained with different scaling of the training set.

|  | Number of training cases | Volumetric DSC | True positive volume fraction | Hausdorff distance 95% |
| --- | --- | --- | --- | --- |
| SegNet ^(A)^ | 42 | 0.82±0.04 | 0.79±0.08 | 10.33±6.40 |
| SegNet ^(B)^ | 40 | 0.82±0.03 | 0.78±0.07 | 9.57±5.07 |
| SegNet ^(C)^ | 39 | 0.81±0.04 | 0.91±0.04 | 10.42±2.47 |

# Section 3: Supplemental Figures

## Figure S1


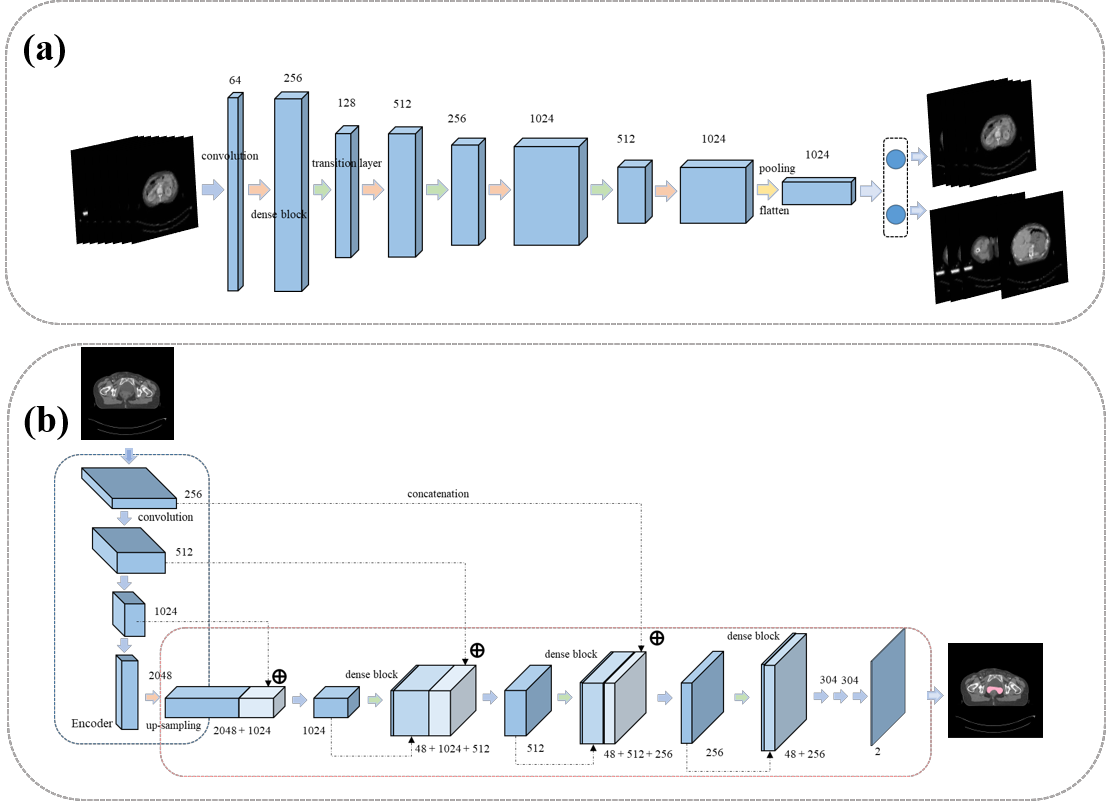


**Fig S1.** Main architecture of the two-stage SegNet: (a) first stage for the identification task and (b) second stage for contour segmentation. The blue cube represents the learned feature maps, and the number next to the cube denotes the corresponding number (channel) of feature maps. Densely connected convolutional blocks and residual blocks are denoted by arrows.

## Figure S2


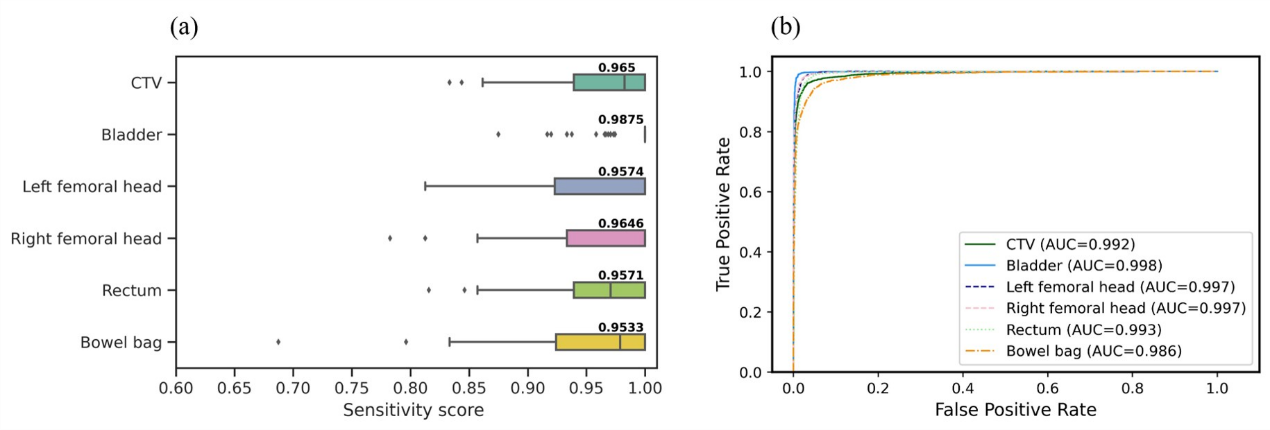


**Fig S2.** Predicted performance of the first stage identification results of six automatic regions. (a) Distribution of sensitivity score for each contour; (b) Illustration of area under the ROC curves.

## Figure S3


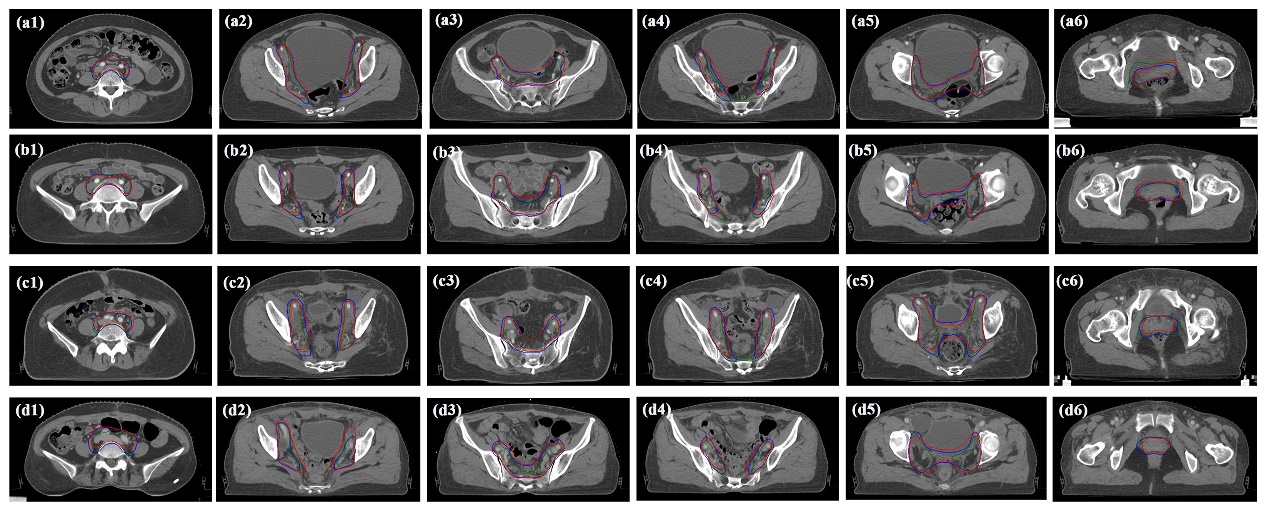


**Fig S3.** Clinical target volume contouring predicted by SegNet and UNet. The blue, green, and red contours represent the CTV produced by SegNet, the CTV produced by UNet, and the corresponding manual annotations, respectively. The first and second columns indicate the areas of the common iliac lymph nodes and the internal and external iliac lymph nodes, respectively. The third and fourth columns present the presacral lymph nodes area and the end of this area, respectively. The fifth and sixth columns indicate the paravaginal tissue and upper vagina area, respectively. The quantitative volumetric DSC scores for cases (a), (b), (c), and (d) by SegNet were 0.86, 0.87, 0.83, and 0.85, respectively. The quantitative volumetric DSC scores for cases (a), (b), (c), and (d) by UNet were 0.84, 0.87, 0.82, and 0.81, respectively.
